# Microfluidic Devices for Forensic DNA Analysis: A Review

**DOI:** 10.3390/bios6030041

**Published:** 2016-08-05

**Authors:** Brigitte Bruijns, Arian van Asten, Roald Tiggelaar, Han Gardeniers

**Affiliations:** 1 Mesoscale Chemical Systems, MESA+ Institute for Nanotechnology, University of Twente, Drienerlolaan 5, Enschede 7500 AE, The Netherlands; r.m.tiggelaar@utwente.nl (R.T.); j.g.e.gardeniers@utwente.nl (H.G.); 2 Life Science, Engineering and Design, Saxion University of Applied Sciences, M. H. Tromplaan 28, Enschede 7513 AB, The Netherlands; 3 Netherlands Forensic Institute, Laan van Ypenburg 6, The Hague 2497 GB, The Netherlands; 4 Co van Ledden Hulsebosch Center, Amsterdam Center for Forensic Science and Medicine, University of Amsterdam, Science Park—Building 904, Amsterdam 1098 XH, The Netherlands; clhc-science@uva.nl

**Keywords:** cell lysis, DNA extraction and purification, PCR, isothermal amplification reactions, microfluidics, chips

## Abstract

Microfluidic devices may offer various advantages for forensic DNA analysis, such as reduced risk of contamination, shorter analysis time and direct application at the crime scene. Microfluidic chip technology has already proven to be functional and effective within medical applications, such as for point-of-care use. In the forensic field, one may expect microfluidic technology to become particularly relevant for the analysis of biological traces containing human DNA. This would require a number of consecutive steps, including sample work up, DNA amplification and detection, as well as secure storage of the sample. This article provides an extensive overview of microfluidic devices for cell lysis, DNA extraction and purification, DNA amplification and detection and analysis techniques for DNA. Topics to be discussed are polymerase chain reaction (PCR) on-chip, digital PCR (dPCR), isothermal amplification on-chip, chip materials, integrated devices and commercially available techniques. A critical overview of the opportunities and challenges of the use of chips is discussed, and developments made in forensic DNA analysis over the past 10–20 years with microfluidic systems are described. Areas in which further research is needed are indicated in a future outlook.

## 1. Introduction

The DNA analysis process at the forensic laboratory sometimes takes days, resulting in the fact that the outcome may have become less relevant to be able to effectively contribute to the initial phase of the criminal investigation, conducted by the police forces [1]. This might give a perpetrator time to eliminate relevant evidence, to disappear or even to commit another crime. On the other hand, it is desired to release innocent suspects quickly from custody [2]. The first hours of investigation are not without reason called the “golden hours”. For these reasons, there is a strong need for relevant information becoming available as quickly as possible [3]. Devices that provide immediate information to police investigators at the crime scene are especially useful, as direct analysis contributes to fast and effective case scenario development.

So-called “lab-on-a-chip” (LOC) technology has the potential to be used for this purpose. An LOC can be defined as a device in which multiple laboratory techniques are integrated in a chip with a footprint of at most a few tens of square centimeters. An LOC comprises an enclosed internal microfluidic channel network with small characteristic dimensions below 1 mm in which reagents can be manipulated on the microscale. The internal volumes of LOCs are within the μL-range, resulting in typical microfluidic benefits, such as rapid heating and fast mixing. As a consequence, LOC devices offer fast analysis time, require minimal amounts of analyte and are portable. Due to sample handling in a sealed microfluidic environment, LOC systems reduce the risk of (cross-)contamination, improve the chain of custody and provide the possibility of direct analysis at the crime scene. LOCs are frequently designed for single use, which will bring benefits with respect to contamination risks and the chain of custody. All of these issues are important within forensic science.

**Figure 1:**
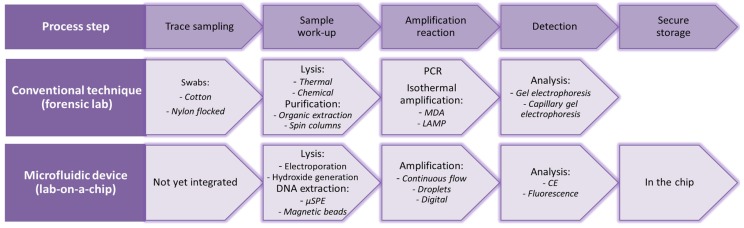
In the top row, the consecutive steps in the process of forensic DNA analysis are listed; within the middle row, the conventional technique with some examples. In the bottom row, microfluidic analogies can be found with some examples.

To get from forensic trace sampling to useful forensic information, several steps can be distinguished, as can be seen Figure 1, in which the conventional techniques, as well as their existing microfluidic counterparts are listed. Ideally, devices are used that can collect a sample from the crime scene, perform sample work-up (lysis and extraction of DNA) and amplify and analyze the DNA. Since the first hours of an investigation are of utmost importance and usually the amount of time available to analyze the crime scene is limited, fast devices are crucial. However, in practice, such fully-integrated devices that can be applied directly at the crime scene are not realized yet. Microfluidic devices developed for point-of-care and clinical applications for cell lysis, sample work-up, PCR and detection/analysis create opportunities, which can also be applied within the forensic field.

In this review, the latest developments for each step will be highlighted. Aspects to improve these microfluidic devices are discussed. Such a critical approach and considerations are a necessity to come to integrated devices for broad use. It will also be discussed at which level these steps have been integrated in a complete microfluidic forensic DNA analysis system. Besides the steps mentioned in Figure 1, also PCR speed records, the current status of DNA analysis on-chip (on the research level, as well as with commercial systems) and the opportunities and challenges of the use of chips are discussed.

## 2. Trace Sampling

**Figure 2:**
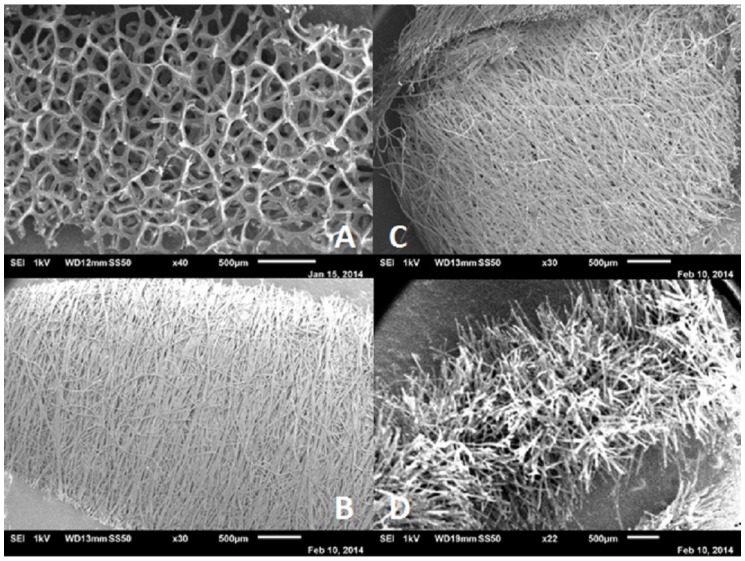
Scanning electron microscope images of several swab materials. (**A**) Foam swab (Sterile Foam Tipped applicator, Puritan); (**B**) rayon swab (155C Rayon, Copan); (**C**) polyester swab (159C Polyester, Copan); and (**D**) flocked swab (3503C 4 ng Floqswab, Copan).

Before DNA analysis can be carried out, the sample must be collected in an appropriate way, such that no contamination takes place and that as much sample as possible is collected. Careful selection of the samples by the forensic investigator is of utmost importance, since only a limited amount of samples can be sent to the laboratory for further analysis [1]. Not only samples from a crime scene, but also reference samples need to be taken in a proper way that is rapid and painless and least invasive for the person (suspect or victim). Typically, swabs are used for this purpose [4]. For the recovery of a sample from the crime scene, several different types of swabs can be used, with different structures, as shown in Figure 2.

In contrast to what is common practice in forensics, most existing micro-devices use cell lysate or pure DNA in a buffer as input material, because microfluidic devices require samples in a liquid solution. Only some commercially available devices, as discussed in Section 8.2, make use of a swab as input. Since flow channels in a chip are typically smaller than 1 mm in diameter, it is very important to prevent clogging, which can, for example, occur due to fibers from a swab. More specifically, new research is required to find out how many fibers swabs release upon forensic use, because this is crucial knowledge to judge their appropriateness for LOC-applications. Loading of the crime scene sample from a swab into a microfluidic device is another challenge that needs more attention in future research.

## 3. Sample Work-Up

The sample work-up usually consists of three steps: cell lysis, DNA extraction and DNA purification [5,6]. The last two steps are often combined when carried out on-chip. After lysis of the cells, the DNA must be separated from the other cellular components, since these can inhibit the amplification reaction [7]. Commonly present inhibitors in forensic samples are ethanol and sodium dodecyl sulfate (SDS) originating from the isolation technique, as well as hemoglobin, calcium-ions, melanin and urea, possibly present in biological samples. These inhibitors can result in a delay in the threshold cycle, reduction in the sensitivity of the detection (especially for larger amplicons) or even failure of PCR amplification [4].

### 3.1. Cell Lysis

To induce cell lysis (on-chip) thermal lysis, electrochemical lysis and mechanical lysis are some of the options [8,9]. Most of the methods and on-chip systems are already used within clinical settings, but are not (yet) applied within forensic applications. In fact, these lysis methods can also be utilized within forensic micro-devices and might give opportunities within forensic DNA analysis.

Thermal lysis is a popular method, as it is simple to integrate with other on-chip steps [8,9]. In the case that PCR will also be performed, the first temperature step in the program can be used to induce the lysis of the cells. Typically temperatures of 94 ${}^{\circ }$C for 2 min are applied for lysis, followed by temperature steps necessary for PCR [10,11]. Wiederkehr et al. used for their on-chip PCR amplification of whole blood an extra temperature step prior to the PCR reaction of 2 min at 98 ${}^{\circ }$C [12]. A micro-device for the capturing and lysis of bacteria cells was developed by Tsougeni et al., in which cell lysis was obtained by applying 93–95 ${}^{\circ }$C for 10 min [13].

A lysis buffer containing surfactants can solubilize the lipid membrane of cells, which is known as chemical lysis [14]. Jen et al. used a lysis buffer with 1% Triton X-100 for their microfluidic chip with an array of micro-wells [15]. The drawback of chemical lysis (on-chip) is the required washing step [9].

Applying high electric field pulses (around 1 kV/cm) will induce an extra transmembrane potential in mammalian cells. Irreversible electroporation is a result of short (around 2 μs) high electric field strength pulses (≥10 kV/cm). The latter technique is used for the inactivation and pasteurization of cells, but also for cell lysis. The advantage is that no chemicals are needed [16]. Lu et al. have developed a microfluidic device for the electroporation of cells. About 74% of the cells could be lysed by applying a voltage of 8.5 V at a frequency of 10 kHz. With this method, cells were lysed in a continuous-flow device in small volumes with a lower power consumption than in conventional larger equipment [14]. Furthermore, Jiang et al. used an electric signal to lyse their cells; a 6.8 V square wave pulse resulted in a lysis efficiency of about 50% [17].

Another lysis method is based on local hydroxide electro-generation, also known as alkaline or electrochemical lysis [9]. The hydroxide ions, by cleaving fatty acids, porate the cell membrane and open it permanently [18,19]. The hydroxide ions will be neutralized afterwards by the protons that are also generated by the electrochemical process [18]. The advantages of this method are the absence of detergents and heating elements [20]. Di Carlo et al. used a voltage of only 2.6 V (43 V/cm) [18]; Nevill et al. used a voltage of 2.5 V (11 V/cm ) [19]; and 10 V was applied for 5 min by Lee et al. in order to achieve successful lysis [20].

Mechanical lysis uses nanostructured filter-like contractions (so-called nano-knives) in a microfluidic channel to lyse the cells. Mechanical lysis is a reagentless method, but only shear and frictional forces are usually not enough to induce rupture of the cell membranes. By integrating sharp nanostructures in the chip, which penetrate the cell membrane, successful lysis can be obtained [21].

Optically-induced cell lysis can also be performed in microfluidic systems. With this technique, a specific cell can be lysed, without damaging the nucleus. By applying an alternating current, a high electrical impedance is present in the lysis zone. When the beam spot illuminates this zone, the impedance decreases substantially, resulting in a nonuniform electric field, which induces a transmembrane potential [22]. Huang et al. applied this principle to lyse cells in their microfluidic device, and for about 78% of the cells, the membrane was lysed [23].

Other conventional (large scale) lysis methods, which are translated to on-chip techniques, are osmotic lysis and ultrasonic or acoustic lysis. These methods are not widely used within (forensic) microfluidics, due to the complexity of the methods. For osmotic lysis, a change in cell medium concentrations and a treatment step prior to the osmotic lysis to weaken the cells is required. With acoustic lysis, heat is generated, which can result in the transmission of energy to the cell medium [24].

A more extensive review of cell capturing and lysis methods in a chip is given by Le Gac et al. [9]. Fox et al. focus on electroporation of cells [16].

An upcoming field is single cell lysis on-chip. Since crime scene samples often contain only a few cells and little amounts of DNA, these methods are of great interest for future forensic developments. Most of the techniques are based on the cell lysis methods as described above, but with an additional cell entrapment system on the chip [25]. Especially single cell electroporation is a popular method [26,27,28]. Jen et al. have developed a chip for chemical lysis of single cells by using an array of micro-wells [15].

### 3.2. DNA Extraction and Purification

#### 3.2.1. (μ)SPE

Solid phase extraction (SPE) by the use of silica is a commonly-used method for on-chip extraction, as it effectively binds the DNA [6,29,30]. Purification of nucleic acids (DNA or RNA) by (μ)SPE consists of a DNA loading, a washing and an elution step [8]. Only nanograms of silica resin in a μSPE device are enough to adsorb and desorb DNA in the pg–ng range [30,31]. Tian et al. have given an overview of possible silica resins and suggest that fully-hydrated acidic silica resins provide the best solid phase for DNA binding, and the complete μSPE procedure takes less than 10 min [30]. A sol-gel with enclosed silica beads can be used to purify the sample in a chip. In theory, silica beads and sol-gels can be used individually; however, the use of only silica beads shows the compression of the particles during use, and sol-gels are mechanically not very stable, which is a drawback of this method. Wolfe et al. suggest to use an acid-catalyzed sol-gel precursor solution combined with silica beads in order to obtain the best results. With this method, they could obtain PCR-amplifiable DNA [32]. Silica beads packed into a glass chip were used by Breadmore et al. to separate the DNA. The beads were immobilized in a sol-gel to provide a stable and reproducible solid phase. The additional advantage of this method is that they could reduce the extraction time from 25 to 15 min by using a pH of 6.1 instead of 7.6 as the loading conditions [33]. Duarte et al. named the DNA extraction on their chip with magnetic silica beads “dynamic SPE”. They could recover more than 65% of DNA from 0.6 μL of blood (lysis of the blood with detergents was performed off-chip), and the concentration of the resultant DNA was above 3 ng/μL. After purification, they performed amplification of the DNA in the chip, and the PCR products were analyzed off-chip [34]. Reedy et al. designed a poly(methyl methacrylate) (PMMA) micro-device and used chitosan to bind DNA in a pH-dependent manner, i.e., binding at pH 5 and release at pH 9. With this chip, they could purify a lysed whole blood sample, which turned out to be PCR amplifiable [7]. By using silica beads in their SPE chip, Zhang et al. obtained a DNA extraction efficiency of about 50% within 15 min [35].

#### 3.2.2. Magnetic Beads

Magnetic beads provide a fast and efficient method for the purification of DNA from a large variety of (small quantity) forensic samples [8,36]. The ChargeSwitch${}^{®}$ PCR clean-up kit from Invitrogen contains magnetic beads to purify the sample from salts, primers, dNTPs and other non-nucleic acid reagents. The charge of the beads depends on the pH of the buffer. At low pH, the beads have a positive charge and will bind nucleic acids, since the nucleic acids have a negatively-charged backbone. By increasing the pH to about 8.5, the nucleic acids can be eluted from the beads. The binding capacity is about 25 μg DNA per 1 mg beads [37]. Hopwood et al. used the ChargeSwitch${}^{®}$ beads to purify the sample in their microfluidic system for rapid forensic DNA analysis [38]. Other commercially available magnetic beads are Dynabeads${}^{®}$. These beads were used by Lien et al. for their reverse transcription-PCR (RT-PCR) micro-device [39]. Phase separation of magnetic beads from picoliter-scale droplets is used, as well, but this is difficult due to the high interfacial tension. Gu et al. used ferromagnetic particles to carry the magnetic beads to overcome this problem [40]. Yang et al. have developed a microfluidic cartridge for sample lysis by inserting a swab head into the lysis chamber. Buccal, saliva and blood swabs were lysed with 1 mL lysis buffer. Three different commercially available lysis buffers were used, all based on magnetic bead chemistry. The lysis efficiency of the cartridge was comparable to in-tube (benchtop) controls [41].

#### 3.2.3. Differential Extraction

Differential extraction on-chip is still a challenge for forensic DNA analysis. Differential extraction means that the male and female DNA fractions must be separated, which is important for sexual assault evidence. In 2006, the group of Landers published an article about cell lysis and DNA extraction of sperm cells on-chip. Their chip is based on SPE with a microchannel partially packed with a sol-gel/bead mixture. The packed sperm cells are subsequently lysed with a lysis buffer, which takes about 15 min. From the purified sample, it was possible to obtain a short-tandem repeat (STR) profile [42]. In 2009, the same group published an article about differential extraction by means of acoustics. They could obtain from mock cases within 14 min highly purified male and female fractions. By the use of ultrasound, the device could selectively trap the sperm cells from a sample that also contained female epithelial cell lysate [43]. Microfluidic Systems, Inc., presented their device at the 9th International Conference on Miniaturizes Systems for Chemistry and Life Sciences in 2005 and patented the design; however, they never published progress or results in a journal. To obtain the separated fractions, they apply sonication to selectively lyse the epithelial cells combined with a filter to separate the epithelial cells from the sperm cells. This method is fully automated and takes less than three hours [44,45]. Landers et al. suggested also another mechanism to separate the fractions. This method makes use of the different physicochemical properties of the two fractions, which result in different sedimentation rates [46].

The use of packed silica beads is a well-established and relatively simple concept, but this technique uses compounds that can inhibit the amplification reaction, which is also the case for sol-gels with silica beads. By using paramagnetic beads, this problem can be overcome, but places restrictions on the device material and dimensions [29]. (μ)SPE is integrated on-chip by several research groups, and by the use of (μ)SPE, whether or not combined with beads, it is possible to obtain a PCR amplifiable DNA extract. However, it is not yet widely applied for DNA analysis in a microfluidic device for forensic applications, which is also emphasized in Section 8 of this review. In recent publications, silica beads were the method of choice for SPE extraction [47,48]. Although magnetic beads can be used as a fast purification method and can be used in chips for forensics as the extraction method, the control of the beads remains complex [38]. Differential extraction is very important in, for instance, rape cases, but the various methods for on-chip extraction have not yet been applied within a micro-device that combines all of the steps of forensic DNA analysis.

## 4. DNA Amplification

Forensic samples are known for the low amount of DNA available, as one cell only contains about 6 pg of DNA [49]. Therefore, it is necessary to perform an amplification reaction to increase the amount of DNA, such that detection and further analysis (e.g., STR profiling) can take place. In order to analyze samples directly at the crime scene, a fast and reliable amplification technique is required. PCR is widely used for STR profiling of DNA samples. Most of the microfluidic devices described in this section are solely PCR chips. Confirmation of the functionality of the chip is carried out by non-specific detection by the use of a fluorescent intercalating dye to evidence DNA amplification or by off-chip gel electrophoresis to determine the amplicon length. Implementation of STR-profiling is still a challenge and is only done by a few research groups and commercial companies, which will be discussed in Section 8. Nowadays, also other methods, such as isothermal amplification techniques, are forthcoming.

### 4.1. PCR

Before amplification, usually a quantification step is included within conventional STR profiling, since the best results are obtained by the use of a specific amount of DNA input. Quantification ensures the maximum efficiency of the amplification reaction, and the (repetitive) analysis of over-amplified samples is prevented [50]. Fluorescent DNA dyes (such as PicoGreen) and slot blot are some of the methods that can be used to detect and quantify DNA within a sample. However, these techniques are not human-specific or are labor intensive and, therefore, became outdated [51]. Real-time PCR is nowadays the method of choice, which is mostly based on the use of the Alu sequences in the human genome. It is even possible, by real-time PCR, to quantify not only the total human autosomal DNA, but also Y-chromosomal and mitochondrial DNA [52].

However, most of the developed micro-devices are designed for either sample work-up or amplification and detection, whereby the input DNA has a known concentration. Therefore, DNA quantification on-chip has not been widely incorporated. In order to overcome the need for quantification, a silica filter or beads can be used, which have a specific binding capacity to prevent an excess of DNA available for the amplification reaction [53,54].

Within the polymerase chain reaction (PCR), the annealing and the extension step can be combined, if primer design allows, an operation often seen within microfluidics [55]. With conventional thermocyclers, a heating and cooling rate of about 2–3 ${}^{\circ }$C/s can be obtained [5].

**Figure 3:**
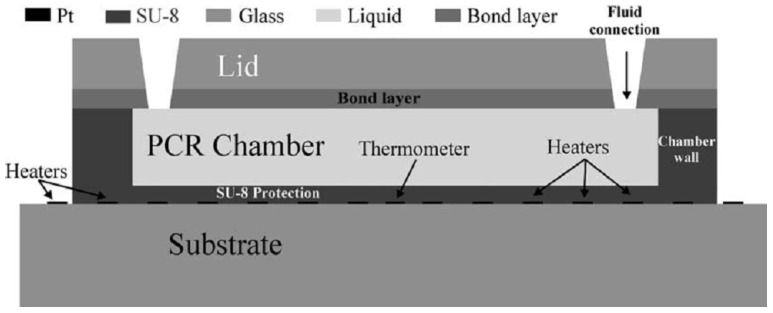
Example of a well-based chip for DNA amplification (reprinted from [56], with permission from Elsevier).

**Figure 4:**
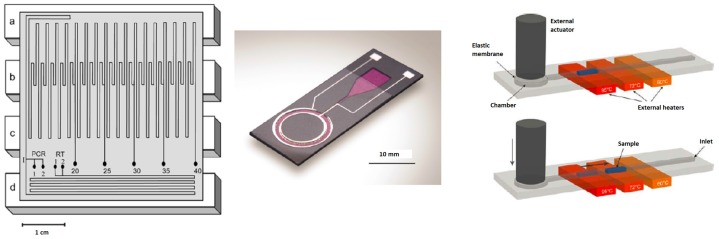
Examples of continuous flow chips with, from left to right, a fixed-loop (reprinted with permission from [58], Copyright 2003 American Chemical Society), a closed-loop (reproduced from [59] with permission of The Royal Society of Chemistry) and an oscillatory chip (reprinted from [60] with kind permission from Springer Science and Business Media) for DNA amplification: Principle of sample shuttling: The PCR reaction is performed inside a straight channel ending in a chamber with a membrane that is deflected to move the liquid sample back and forth over three constantly-heated regions. Actuation and heating is done externally, so that the chip can be kept as simple as possible.

In the past decade, a wide variety of microfluidic devices for DNA amplification has been developed. In microfluidic devices, heating and cooling rates of at least 10–50 ${}^{\circ }$C/s can be obtained [5,56]. The micro-devices can be divided into two main types: well-based and continuous-flow PCR chips [57]. Examples of these different types of chips can be seen in Figure 3 and Figure 4 [56,58,59,60].

For continuous-flow chips, the sample must be moved through fixed temperature zones to perform thermal cycling. In contrast to well-based systems, only the sample needs to be heated and cooled, and not the entire chip. In 2009, Zhang et al. gave an overview of microfluidic DNA amplification devices that were developed at that time. Most of the devices were continuous-flow PCR chips with a fixed-loop design [57]. An overview of the variety of well-based and continuous-flow PCR chips and their characteristics can be found in Table 1. Besides the number of cycles, also the type and/or length in base pairs (bp) of the amplicon is given and the total cycling time.

The upcoming field of interest is the principle of PCR in droplets. By the use of droplets, the analysis time can be shortened, and each droplet can be seen as an individual reaction volume [57]. In the following subsection well-based chips, continuous-flow designs, as well as PCR within droplets will be discussed.

#### 4.1.1. Well-Based Chips

In a well-based PCR system, the well, or even the complete chip, is sequentially cooled and heated during the PCR temperature cycles. The disadvantage of such a system is the long cycling time, due to the large total thermal mass of the system [57].

El-Ali et al. have modeled and developed a well-based chip that can reach heating and cooling rates up to 50 and 30 ${}^{\circ }$C/s, respectively, using integrated heaters. The chamber can contain a volume of 20 μL and is made of SU-8 on a glass substrate [56].

A chip with nanoliter wells for real-time quantitative PCR amplification has been developed by Liu et al. The polydimethylsiloxane (PDMS) chip consists of 100 wells of 120 nL with pre-loaded dried primer pairs. Cycling was carried out with a thermoelectric cooler, and the fluorescent dye EvaGreen was used for the detection of the PCR product [61].

Zhang et al. have developed a well-based chip with a nanoliter droplet array for RNA amplification. By using a TaqMan${}^{®}$ probe, the amplification process could be monitored in real-time [62].

#### 4.1.2. Continuous-Flow Chips

Continuous-flow chips are divided into fixed-loop, closed-loop and oscillatory chips. Each type of method has its own advantages and disadvantages, which will be discussed below.

##### Fixed-Loop Chips

A fixed-loop system contains zones with different temperatures through which the sample is moved. The number of thermal cycles is fixed by the amount of meanders in the design, and the timing of each step is typically controlled by the length of the meander in a specific temperature zone [57].

Kopp et al. have developed the first continuous-flow PCR chip. By changing the flow rate, the total reaction time varies between 90 s and 18.7 min, although a faster protocol results in less PCR product [64]. A PMMA chip has been developed by Qi et al. to amplify an amplicon of 990 base pairs. The chip, based on the design of Kopp et al, consists of 20 thermal cycles for a PCR mixture of 10 μL [66].

Obeid et al. have developed a continuous-flow chip (Figure 4 on the left) for DNA and RNA amplification in combination with laser-induced fluorescence (LIF) detection and SYBR Green I. The denaturation, annealing and extension steps were carried out in time ratios of 4:4:9. The product can be analyzed after 20, 25, 30, 35 and 40 cycles, which gives with a flow rate of 1.26 μL/s and a total cycle time of 5, 6, 7, 8 and 9 min, respectively. The smallest amount of DNA they could detect was 50 fg [65]. In another publication, Obeid et al. showed that the complete process from sample injection to product collection after amplification takes 35 min. By using hand-driven injection, the reaction of a 10-μL sample could be completed (30 cycles) within 6 min [58].

##### Closed-Loop Chips

In a closed-loop chip, the sample must be moved through a fixed circuit, whereby the number of thermal cycles can vary [57].

West et al. have developed a closed-loop chip (Figure 4 in the middle) in which a cycle time of three minutes or less is possible. Movement of the fluid was performed by using magnetohydrodynamic actuation using a 1-kHz AC signal. A two-step PCR reaction was carried out successfully, although the authors of the article recommend a three temperature zone design to provide more flexibility [59].

The heating required for the PCR was used by Chen et al. to induce fluid motion by Rayleigh–Bénard convection, such that there was no pump required. They developed a Teflon tube loop-based reactor with three heating zones. The reactor loop is put at an angle with respect to the horizontal plane to create convection. Successful amplification of a 305- and a 700-bp fragment could be carried out within 35 cycles with a total reaction time of 73 min (including the final extension of 7 min). However, the shorter amplicon showed a higher amplification efficiency than the longer amplicon [70].

##### Oscillatory Chips

The number of cycles can be varied in an oscillatory system. The different chambers on the chip are held at different temperatures, and the sample is shunted back and forth between these chambers [57].

Bu et al. developed a design with a bi-directional peristaltic pump to shunt the sample back and forth between three chambers with different temperatures. A droplet of only 1 μL could be operated, which can be heated and cooled to the desired temperature in less than 1 s with this design using integrated heaters. However, no real PCR chip has been made; Bu et al. only proposed a design and a theoretical evaluation in their article [71].

By shunting a sub-microliter sample back and forth between three temperature zones (Figure 4 on the right), Frey et al. could combine the cycling flexibility of a chamber-type device with the fast principle of continuous-flow devices. The chip was designed as a disposable device for quantitative PCR reactions. Successful real-time amplification could be carried out in less than 5 min [60].

#### 4.1.3. PCR Speed Records

For several years, there has been an on-going competition regarding the record for fastest on-chip amplification. Giordano et al. claimed in 2001 that their well-based chip could perform DNA amplification within 240 s. They used a 500-bp amplicon, and a PCR product was observed after 15 cycles (15 s per cycle). Temperature control was carried out with a thermocouple within the 1.7-μL PCR chamber [74]. In 2006, Neuzil et al. published two papers about their ultra-fast real-time well-based PCR micro-device [75,76]. One thermal cycle was conducted in 8.5 s, which means they could complete 40 cycles in 340 s. However, the length of their amplicon was only 83 bp [75]. In 2011, Fuchiwaki et al. claimed to have the fastest PCR chip, which makes use of continuous-flow, in the world. The 40 cycles could be completed, with a fluorescence level of 15%, in only 120 s. However, an 80% amplification/yield was obtained after 600 s [77]. Son et al. achieved similar results (30 cycles within 5 min) by using a thin Au film and light emitting diodes as a light-to-heat convertor and a heat source, respectively. A 98-bp amplicon could be successfully amplified with this photonic system [78]. They reduced the amplification time to 4 min for 30 thermal cycles for a 116-bp amplicon with an optofluidic cavity PCR device [79]. Most records were obtained by the use of a relatively short amplicon and optimal conditions (e.g., high DNA input and optimal primer concentrations). Therefore, it is questionable if such timescales are realistic for forensic applications, since with STR profiling amplicons can be 200 bp in length or even more and usually real crime scene samples contain a low amount of DNA.

#### 4.1.4. PCR in Droplets

**Figure 5:**
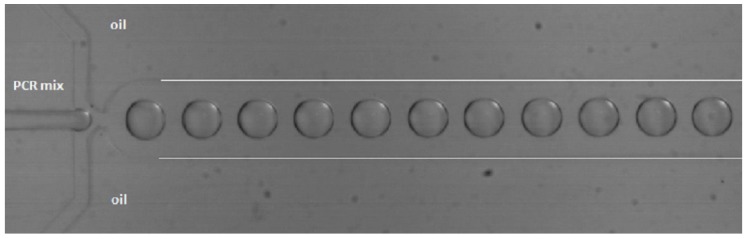
Droplet generation in a flow focus chip from PDMS with a channel width and height of 200 and 104 μm, respectively. The flow rates of the water and oil phase were 0.1 and 5 μL/min, respectively. The white lines indicate the channel walls.

Droplet-based microfluidics is an upcoming field of research. Reactions can be performed in water-in-oil droplets (biphasic) in microchannels, whereby each droplet functions as an independent reactor (pico- to nano-liter size), as can be seen in Figure 5. Droplet-based microfluidics offers several unique advantages when compared to conventional and single-phase continuous flow techniques, since less sample and reagents are required and no interaction with the channel walls takes place, because the droplets are isolated by the carrier fluid. High surface-to-volume ratios lead to shorter heat and mass transfer times and increase the mixing efficiency, making the system faster, and by using meandering channels, mixing can be even more efficient. The droplets are separated sample plugs, which prevent (cross-)contamination [57,80,81,82,83,84].

Several groups have developed a chip for DNA (and RNA) amplification in nano- to pico-liter droplets. An overview of several chips for droplet amplification and their characteristics is given in Table 2. Besides material choice, the number of cycles (with type/length of the amplicon) and the detection method, the droplet size is given.

A numerical and experimental study of a droplet-based PCR chip to carry out a two-step PCR reaction was carried out by Mohr et al. The thermal conductivity and density of the aqueous and oil phase should not change too much in the PCR temperature range to ensure optimal thermal properties. Mohr et al. showed numerically that the variation is less than 3% and 4% for water and oil, respectively, when temperatures of 60 and 95 ${}^{\circ }$C are used [86].

Beer et al. have developed a microfluidic real-time PCR instrument for generating monodisperse microdroplet reactors, including thermal cycling for PCR and detecting real-time amplification in the individual picoliter droplets. Before thermal cycling of the PCR starts, an off-chip valving system stops the flow of droplets, after which the droplets remain stationary during the entire PCR reaction. The reactor size is six orders of magnitude smaller (pL instead of μL) than commercial real-time PCR systems, and an approximately 56% cycle reduction can be acquired. Only 18 cycles are required for single-copy real-time detection on the chip by the use of TaqMan-based FRET probes [85]. The same research group also developed a picoliter droplet chip for RNA isolation followed by RT-PCR. The process of amplification can be followed in real-time by fluorescence. Only 23 cycles are needed for single-copy reverse transcription from RNA, amplification and detection on-chip using TaqMan-based probes [93].

Kiss et al. have developed a continuous-flow device in which the oil stream guides the droplets through different temperature zones within 55 s. Within 35 min, they can detect a 245-bp *Adenovirus* product with one template molecule per 167 droplets, which is as low as 0.003 pg/μL [88].

Hatch et al. have developed a digital droplet chip, which can generate more than one million monodisperse 50 pL in 2–7 min. The two-step PCR takes about 65 min with a total of 40–45 cycles. By using FAM probes, the amplification reaction can be monitored in real-time [90].

A radial PCR device with a hot zone for denaturation of the DNA and an annealing/extension zone has been developed by Schaerli et al. The amplification efficiency of a short 85-bp amplicon is about 20-fold higher than a long amplicon of 505 bp. With the design, a droplet formation frequency of 15 Hz can be obtained with a residence time of 29 s per cycle [87].

The agarose droplet method, developed by Yang et al., can be used for single molecule emulsion (RT-)PCR and amplicon trapping. The agarose droplets are gelated to form agarose beads after amplification. Ultra-low gelling agarose was used with a melting and a gelling point of 56 ${}^{\circ }$C and 16 ${}^{\circ }$C, respectively. This means that the agarose is in the liquid phase when the temperature is ≥16 ${}^{\circ }$C, and once solidified, the beads stay solid till ≤56 ${}^{\circ }$C [89,91,94]. Geng et al. designed a method for multiplex STR-typing at the single-cell level by the use of agarose droplets and a microfluidic device. The method is based on several consecutive steps (such as two PCR steps), which are not all performed on-chip. The total analysis time, from cell lysis up to capillary electrophoresis (CE) detection, is 22 h [92,95].

#### 4.1.5. dPCR

Digital droplet PCR is the latest alternative method for the conventional real-time quantitative PCR technique. With limiting dilution, the sample can be divided into separate reaction chambers, whereby position statistics can be applied [96]. In 1992, Sykes et al. suggested a method to quantitate the total number of initial targets that are present in a sample. The sample, together with the PCR mixture, is divided into a large number of separate small volume reactions. Some of these small reaction volumes will contain a target molecule, and others will be empty. A reaction with a target molecule can be counted as ‘1’ (positive) and without target molecule as ‘0’ (negative), and therefore, the total number of positive reactions can be used for the quantification of the original sample. By separating the original sample into a high amount of small volumes, Poisson statistics can be used [97].

The well-based chips of Liu et al. and Zhang et al. are also digital PCR devices for real-time quantitative PCR [61,62]. The one million droplet array of Hatch et al. is an example of a micro-device that makes use of droplet PCR, as well as digital PCR [90]. White et al. used the digital PCR chip from Fluidigm in combination with TaqMan PCR for the absolute quantification of sequencing libraries [98]. Furthermore, Sanders et al. used this microfluidic device to perform quantitative PCR with less than 200 DNA copies, and they obtained reproducible results with high precision [99]. A droplet digital PCR device has been used by Wang et al. to perform absolute quantification of microRNA, which is lung cancer related [100].

### 4.2. Isothermal Amplification

For isothermal amplification reactions, there is no need for thermal cycling, which makes the (micro-)systems for isothermal amplification simpler and less energy-consuming, which is attractive for portable battery-operated instruments. Another characteristic of isothermal reactions is that the rate of the enzyme activity is the limiting factor instead of the rate of thermal cycling, as with PCR. Miniaturized systems can be composed of one microchamber, and no fluid motion is required during the amplification [101].

Just as with PCR, it is possible to perform real-time quantification of amplification products with fluorescent DNA probes or intercalating dyes. Whereas several inhibitors are known for PCR (e.g., heme, heparin, urea and acidic polysaccharides), isothermal techniques do not suffer from many of these inhibitors [102].

Enzymes that perform strand displacement are used within isothermal amplification methods. Therefore, no heating step is necessary to denature the DNA from double-stranded to single-stranded DNA [102]. φ29 DNA polymerase is such a polymerase with strand displacement activity. Moreover, φ29 shows proof-reading activity and is capable of generating very long synthesis products [103].

Loop-mediated isothermal amplification (LAMP) and multiple displacement amplification (MDA) are examples of isothermal reactions. An overview of these and some other isothermal techniques, namely helicase-dependent isothermal DNA amplification (HDA), rolling circle amplification (RCA) and strand displacement amplification (SDA), is given in Table 3 [101,102,104]. These isothermal techniques are mentioned in the review of Auroux et al., who only mentioned two on-chip examples, without information on the integration of these techniques into microfluidic devices [55]. In 2008, Gill et al. described several isothermal amplification techniques in their article. They explained among others the methods SDA, RCA, LAMP and HDA [104]. In 2011, Asiello et al. gave an overview of several isothermal amplification methods, such as LAMP, HDA and recombinase polymerase amplification (RPA), and their applications in miniaturized systems [101]. A critical review about isothermal techniques for point-of-care applications was given in 2012 by Craw et al. [102]. Recently, Safavieh et al. reported an extensive overview of microchip and micro-device technologies for amplification by LAMP [105].

An overview of various isothermal amplification chips is given in Table 4. Below, some details of the two most studied methods, LAMP and MDA, are given.

#### 4.2.1. LAMP

LAMP is an isothermal nucleic acid amplification method with high specificity, efficiency and speed [117,129]. When Bst polymerase is used, the reaction can be carried out at 60–65 ${}^{\circ }$C [106,129,130]. By using this technique, the target sequence is amplified three-fold every half ‘cycle’ [130]. Within 1 h, a few copies of DNA can be amplified by LAMP up to a detectable amount [129]. Mori et al. claimed that 10–20 μg is produced within 30–60 min [106], and Notomi et al. obtained 10${}^{9}$ copies in less than an hour [130].

Four or six specially-designed primers are required, as well as a polymerase with strand displacement activity (e.g., Bst polymerase). The loop primers (two) increase the speed of the (start of) the reaction, but are not essential [106,129]. The two outer primers are only used in the first steps; thereafter, only the inner primers are used. The correct primer design for the LAMP reaction is complicated and challenging [130]. The product, a mixture of stem-loop DNAs with various sizes of stem and cauliflower-like structures, can be detected by real-time measurement of turbidity, since pyrophosphate ions are a byproduct of the reaction, which is visible as magnesium pyrophosphate in the reaction mixture [106,130,131]. The relation between the turbidity and the amount of DNA is linear [131]. A positive reaction is visible as a white precipitate and can be observed by the naked eye [107]. Another option is to use SYBR Green I as the DNA stain, with a detection limit of 210 copies/mL according to Cai et al. [130,132]. Deguo et al. worked on the detection of *Salmonella* and have reported that the LAMP reaction is only effective when the template is pure. In fact, when there are inhibitors present, the sensitivity of PCR is higher [133].

Digital LAMP was conducted by Gansen et al., and they claimed that amplification in test tubes required less than 1 h. This technique can be used to quantify absolute concentrations of DNA in a biological sample with sample volumes less than 2 μL [117]. Luo et al. designed a microfluidic device for the multiplex real-time quantitative differentiation of bacteria by the use of LAMP. The chips contain eight isolated electrochemical chambers with a total volume of about 20 μL. The whole differentiation process takes about 45 min, and the end of the reaction is electrochemically detected with a decreasing redox current. Furthermore, SYBR Green dye was added after the amplification for detection by the use of UV light [134].

A forensic DNA test with the use of the LAMP reaction has been developed by Watthanapanpituck et al. to determine if a sample is of human origin. The primers developed are human-specific and amplify the human cytochrome b. The product was analyzed with ethidium bromide (EtBr)-stained agarose gels and colorimetric detection with non-cross-linking gold nanoprobes. The test could identify human DNA from several biological samples, such as fresh blood, semen and saliva. The human samples showed a positive signal, whereas the samples from chimpanzee, orangutan, mouse, dog, cat and some other animals did not amplify [135].

#### 4.2.2. MDA

MDA is also known as whole genome amplification (WGA) and is a strand displacement method. The method can be used to amplify circular DNA, as well as linear DNA and makes use of random hexamer primers and φ29 DNA polymerase. Samples with at least 1 ng of DNA (in not more than 1 μL) are normally incubated overnight (16–18 h) at 30 ${}^{\circ }$C with a termination step at 65 ${}^{\circ }$C for 10 min, as suggested by the manufacturer [108,136]. However, Dean et al. observed a plateau reached after 4–6 h [108]. Furthermore, Kumar et al. could generate 4–7 μg of DNA from a single human cell within 4 h [137]. MDA shows a self-limiting reaction with a plateau when around 0.7–10.0 μg/L is reached [121]. The processivity of φ29 is higher than 70 kb, which is the highest known for any polymerase. This allows the replication of the whole genome with only one binding and priming event necessary. There is no need for unwinding proteins (such as helicase), since φ29 shows strand displacement activity. The DNA amplification with φ29 is a continuous process, which is exponential and completely isothermal [108,138]. Although not necessary, it is possible to denature the DNA by heating the template mixture at 95 ${}^{\circ }$C for a few minutes, after which the polymerase needs to be added, as φ29 will be heat inactivated at temperatures of 65 ${}^{\circ }$C [108,136]. In comparison to Taq DNA polymerase, φ29 shows an excellent proofreading activity and a lower misincorporation rate [138]. Twenty to 30 μg of product can be generated from as few as 1–10 copies of DNA; the lysate does not have to be purified, and therefore, it is possible to carry out MDA from samples such as crude whole blood and tissue culture cells [108,136]. Despite the isothermal advantage, MDA has two main drawbacks: non-specificity due to the random primers (which can also lead to the synthesis of the DNA contamination or primer dimers) and uneven representation of the template due to amplification bias [121].

Although Asiello et al. do not describe the MDA reaction in detail in their article, they claim that microfluidic devices based on MDA could be useful in forensics and single cell sequencing [101]. Marcy et al. studied the amplification by MDA from a single cell, and they could obtain sufficient DNA for sequencing [121]. Ballantyne et al. showed that it is possible to use MDA to amplify genomic DNA from small amounts of template, 5 pg–1 ng, for downstream STR multiplex genotyping. The amplification success is increased when a small amount is mixed with a second larger DNA sample. Therefore, the use of molecular crowders, additional DNA or polymers, such as PEG, increases the success rate of MDA, producing more detectable alleles in STR-genotyping [139]. The same group also investigated two different methods for WGA: the GenomiPhi and the GenomePlex kits. The two kits were tested on STR-genotyping of low copy number (LCN) and degraded DNA samples. They showed that by using WGA, the quality and quantity of DNA can be increased and that it has the potential to improve STR-typing from difficult samples in forensic casework. Both kits could amplify the LCN DNA, but only GenomiPhi showed an increase in profiling success. The profiling success from digested DNA was improved by both kits [140].

Although fast well-based and continuous-flow PCR chips are realized, with amplification times under 5 min, these systems are not very suitable for forensic applications. In forensic casework, the samples are not as ideal as in the researched conditions, with pure DNA as input and very short amplicon lengths (under 100 bp). In order to reduce the amplification time, on-chip PCR in droplets and/or isothermal amplification techniques are promising alternatives, due to the many advantages, such as no interaction with the channel walls, increased mixing efficiency, shorter heat and mass transfer times (for droplet PCR) and/or no thermal cycling required (for isothermal amplification).

## 5. Detection

There are various methods for the detection of DNA, as already shown in Table 1, Table 2 and Table 4. Absorbance detection is an option, but the majority of the techniques using on-chip are based on fluorescence detection, sometimes combined with CE. Three main principles result in the occurrence of fluorescence: an (intercalating) dye to stain the DNA, fluorescently-labeled deoxynucleotides (dNTPs) and the incorporation of a fluorescent dye into the amplicon with a labeled oligonucleotide primer. An advantage of dye-labeled primers is that they can be used in multiplex assays, such as STR analysis [4]. Intercalating dyes, such as SYBR Green and EvaGreen, are sequence independent. For specific detection, hydrolysis probes, such as TaqMan probes, or conformation probes (molecular beacons) can be used [5,55].

### 5.1. Absorbance Detection

To quantify dsDNA, the absorbance at 260 nm can be measured by UV-spectrophotometry, which is a fast and simple method [51]. The drawback of this technique is that such measurements are rather insensitive (below 5 ng/μL, measurements are not accurate, which is a typical concentration of a forensic sample) and influenced by the contribution of nucleotides and single-stranded DNA or contaminants (such as proteins or phenol) [51,52,141]. Although the absorbance measurement can still be used as the indicative method, for forensic applications, fluorescence methods are used for the detection and quantification of DNA, such as nucleic acid stains and nucleic acid labels [51]. Another option to monitor DNA amplification with absorbance detection is by using pH-sensitive dyes. When a deoxynucleotide triphosphate (nucleotide) is incorporated into the new DNA strand by the DNA polymerase in an amplification reaction, a hydrogen ion is released. This results in a drop of the pH of the amplification mixture. Tanner et al. used phenol red, cresol red, neutral red and m-cresol purple to show the pH change within LAMP, which is visible by the human eye after 30 min of incubation at 65 ${}^{\circ }$C. Furthermore, visual detection of PCR (1287-bp amplicon and 40 cycles) with phenol red and SDA with all of the above-mentioned dyes is possible [142]. Rodriquez-Manzano et al. used an unmodified camera phone to monitor the amplification of λDNA and hepatitis C viral RNA with LAMP carried out on a chip. They used Eriochrome Black T to detect the color change going from purple to blue upon amplification by using the green/red ratiometric value [143].

### 5.2. Fluorescence Detection

#### 5.2.1. DNA Dyes

A DNA dye stains all of the DNA present in a sample, and therefore, only one extinction and emission wavelength can be used. Differentiation between different amplicons is thus not possible, which is a drawback of this method. There are three main classes of nucleic acid stains: intercalating dyes (e.g., EtBr and PI), minor-groove binders (e.g., DAPI and Hoechst dyes) and other nucleic acid stains (e.g., acridine orange) [144].

In the past, EtBr and PI were often used, which belong to the group of classic intercalating dyes. However, the cyanine dyes are much more sensitive and far less mutagenic than a classic gel stain, such as EtBr [144]. An additional advantage is that most unsymmetrical cyanine dyes, such as SYBR Green and EvaGreen, have a low background fluorescence, a high binding affinity to DNA and a high fluorescent quantum yield [145].

Dyes, such as SYBR Green and EvaGreen, are already widely incorporated in on-chip analysis of DNA, as can be seen in Table 1, Table 2 and Table 4.

#### 5.2.2. Fluorescent dNTPs

Fluorescently-labeled dNTPs will be incorporated in the DNA during the amplification reaction (usually PCR). This type of DNA detection is used in Sanger sequencing, but with the upcoming other (whole genome) next generation sequencing techniques, this is not widely used anymore [146,147].

#### 5.2.3. Fluorescent Primers

To detect a complementary target sequence, a fluorescent-labeled primer or probe can be used. Fluorescent-labeled primers are more expensive than DNA dyes, but more than one color (dyes with different emission wavelengths) can be used. Therefore, amplicons can be separated by using different colors for different primers. Most forensic STR kits use four to five dyes in combination with CE detection and analysis [4].

Liu et al. used for their chip, the PowerPlex${}^{®}$ 16 System, a system that allows co-amplification and three-color detection of sixteen loci [148,149]. Other used kits on-chip, among others, are Profiler Plus${}^{®}$ [47], the PowerPlex${}^{®}$ 18 Fast System [150], AmpFlSTR${}^{®}$ MiniFiler™ and AmpFlSTR${}^{®}$ Identifiler${}^{®}$ amplification kits [48].

### 5.3. Capillary Electrophoresis

In order to generate an STR profile, separation and detection of the DNA fragments must take place. As Pascali et al. indicate in their review from 2011, an important field of interest within forensics is the development of microfluidic devices for CE in order to obtain an STR profile [151]. Mitnik et al. developed a glass chip for the detection of STR fragments by the use of electrophoresis in combination with laser-induced fluorescence. Within 20 min, a single-base-pair resolution of 0.75–1 can be observed for eight loci [152]. A CE micro-device for mini Y STR genotyping is developed by Chen et al. Although a high background of female DNA was present, they could perform the analysis with only 25 pg of male DNA. The seven loci could be separated and detected in the 7 cm-long separation channel by laser-induced confocal fluorescence microscopy [153]. Date-Chong et al. could obtain a full STR profile from a reference sample with their system (RapidHit). The software of the system had several built-in quality flags, which appear, e.g., in the case of an inconclusive homozygote allele, intra-locus imbalance or out of allelic/locus marker range. Of all of the reference buccal samples, 50% passed the two criteria of generating a full profile, without any quality flag that would require manual review/editing [154]. A micro-device with a 1.5-cm channel was used by Aboud et al. for the separation of seven loci (MP7 STR kit) within 80 s with a sizing precision of ±0.21 bp [155].

For on-chip analysis, DNA dyes, such as SYBR Green I or EvaGreen, are widely used. Detection by a fluorescent (intercalating) dye is simple and fast, but these dyes are non-specific, and multiplexing is not possible. For specific detection, fluorescent primers or probes are usually the method of choice. In order to obtain an STR-profile, CE needs to be combined with a fluorescent primer or probe, which makes chip design and detection more complicated. Integration of the detection step into a chip is not always easy, which is also reflected in Table 1, Table 2 and Table 4, by the amount of chips that make use of off-chip detection.

## 6. Secure Storage

Extracted DNA is usually stored at −20 ${}^{\circ }$C or −80${}^{\circ }$C [156]. The Netherlands Forensic Institute has a state-of-the-art integrated storage freezer from Hamilton for extracts at −80 ${}^{\circ }$C (up to 80 years), conforming to the legal terms of the Dutch law [157]. There is a need for secure storage at room temperature, since freezing of samples is costly and there is a serious risk of failure. Anhydrobiosis, whereby water is replaced by other compounds, such as trehalose, is forthcoming. Frippiat et al. showed that with commercially available kits, it is possible to store the DNA extracts for more than six months at room temperature and that the samples were preserved from degradation [156]. Furthermore, Lee et al. evaluated a commercially available storage medium that can be used at room temperature. They concluded that no significant difference could be seen between liquid frozen DNA samples and samples stored in the medium at room temperature. For low concentrations and long-term storage, the DNA recovery was even higher by using the storage medium, which makes it a promising method for the secure storage of forensic DNA samples [158].

Nevertheless, the secure storage has not yet been widely integrated in conventional DNA analysis techniques. By using chips, the sample stays in an enclosed environment, whereby the chain of custody is ensured and the risk of (cross-)contamination minimized. The developments of techniques for on-chip secure storage are of utmost importance for the forensic field and can create opportunities in comparison with conventional techniques. Integrated sample coding contributes to the chain of custody, and follow-up analysis of the DNA in a forensic laboratory can be carried out. It is possible to secure the end product of the analysis performed at the crime scene, as well as an untreated part of the sample (e.g., before the sample work-up) by dividing the input sample into a part that is directly analyzed and a part that is stored on-chip for further analysis in a forensic laboratory. Not much research has been performed yet on the secure storage on a chip of biological samples, although for forensics, it is really important to store the sample in a secure way.

## 7. Chip Materials for DNA Analysis

Most microfluidic chips are made of silicon or glass, although during the last decade, the use of plastic substrates (for instance PDMS or PMMA) has become very popular [159]. For forensic applications, chips are only for one-time use, thus disposable, and can therefore be made of an inexpensive material, as long as it fulfills all requirements. The selection of materials is not always straightforward, since the material itself can negatively affect the steps in the process of DNA analysis, such as PCR and detection.

Glass is widely used because of the favorable optical (transparent) and electrical properties (insulating, which is useful for electrophoresis on-chip). In order to minimize the absorption of the PCR components on glass, the material must be coated or passivated [29,34].

Silicon is also widely used for chips due to its good thermal conductivity, which makes it suitable for the fast heating and cooling required in PCR cycling. However, because it is a semiconductor, it cannot withstand the high voltages required for CE. Furthermore, it is not transparent to the UV-VIS light needed for detection. Untreated silicon is known to inhibit the PCR reaction [160,161]. Cho et al. performed a small literature study and concluded that most static PCR chips are made from silicon. Furthermore, glass is used, but silicon is preferred due to its high thermal conductivity (although thermal isolation is necessary) [161].

Plastic devices involve fast fabrication (mostly based on molding) and are very suitable for SPE and CE on-chip [29,34]. The low costs make plastic chips suitable as disposables, which reduces maintenance and especially cross-contamination issues [67,162]. Plastic devices are attractive due to the bio-compatibility, but have a low thermal conductivity [161]. For continuous-flow devices, glass or plastic is more used, since a low thermal conductivity is desirable for the realization of different temperature zones in the chip [67]. Therefore, many microfluidic devices for biological fluid analysis are made from PDMS or PMMA [18,19,39,88,163,164], although glass or silicon is also utilized [31,58,65,152,165].

Many microfluidic devices for droplet generation or microfluidic DNA amplification are made of PDMS [166,167,168,169,170]. The benefit of using PDMS is the fact that the surface of the channel is hydrophobic. Aqueous droplets in oil are more easily formed in channels with a hydrophobic surface [171]. Next to that, PDMS is bio-compatible, transparent and easily moldable [172]. Due to its permeable nature, uncoated PDMS might show inhibition of the amplification reaction by adsorption or absorption of components in the amplification mix, especially the enzyme. This problem can be solved by coating of the PDMS with BSA [173]. To prevent evaporation, also parylene C can be used to coat the chip [174,175]. In case of water-in-oil systems, the template DNA present in the aqueous phase cannot adsorb to the walls of the chip due to the hydrophobic properties of the oil [176].

The material choice highly depends on the desired functionalities of the chip. Optical and electrical properties are important characteristics to take into account. The selected material should avoid absorption of components in the amplification mixture or inhibit the amplification reaction. An important aspect of chips for forensic applications is that they are disposable, to ensure no contamination can occur. Furthermore, production costs and material pricing are important factors.

## 8. Chips with Integrated Functionality

On the research level, several microfluidic devices (mostly continuous-flow) for DNA analysis with two or more steps, as depicted in Figure 1, integrated into one chip are reported. Table 5 shows various integrated chips and their characteristics. The applied methods for lysis, extraction and detection are given. The challenge is to combine all of the steps of the complete DNA analysis process, from sample input to analysis and secure storage of the sample. Nowadays, a few commercial systems are available, although their deployment is still limited. In the following subsections, various research chips, as well as some commercial chip-based systems are discussed in detail.

### 8.1. Research Chips

A miniature analytical thermal cycling instrument (MATCI) has been developed by Northrup et al. By the use of silicon reaction chambers, several amplicons could be successfully amplified and detected in real-time with EtBr. The heaters are integrated in the system, and the whole PCR and detection setup fits into a briefcase [183].

Lee et al. have made a microfluidic device to carry out cell lysis and DNA amplification by combining an electroosmotic pump, an active micromixer and a temperature control system on-chip. The cells are thermally lysed and then moved to the PCR section by the use of an electroosmotic pump. The complete PCR is carried out within 27 min with a total sample volume of 15 μL [11]. A module for lysis, PCR amplification and detection by CE are combined by Jha et al. They developed a glass chip for the genetic analysis of various cell types and λphage DNA, with amplicon sizes of 100–595 bp. The amplification time depends on the used flow rate. For a flow rate of 5 μL/min, the retention time in each of the three zones is about 30 s (which makes a total amplification time of about 37.5 min). The total time, from lysis till detection, is over 40 min with 22 min for cell lysis and 15–20 min to detect the amplicon with CE-AD (capillary electrophoresis with amperometric detector) [180].

Hopwood et al. have developed a microfluidic system for rapid forensic DNA analysis by means of which it is possible to obtain an STR profile from a suspect (or victim) within 4 h. The chip combines DNA purification, PCR amplification and separation of the amplicons by CE in combination with laser-induced fluorescence (LIF) detection. As the input material, cell lysate is used from a buccal swab [38]. Over time, several improvements have been made to this MiDAS system (miniaturized integrated DNA analysis system). An upgrade is that not the lysate, but the swab head can be used as the input via the integrated swab sample lysis module. In this system, the complete process from lysis up to STR detection takes more than 2 h [184]. Furthermore, Bienvenue et al. designed a chip for forensic DNA analysis via PCR amplification of STR fragments. A conventional thermocycler is used for the amplification reaction on-chip with a volume of 1.2 μL. The cells were chemically lysed off-chip with proteinase K and fuanidine and introduced into the micro-device for the purification by means of an SPE channel filled with silica beads. Detection and analysis were also done off-chip, with conventional CE [47]. Within 42–45 min, the PCR product could be obtained from a sample by the use of a PMMA chip for DNA extraction and PCR amplification. A part of a swab can be loaded into the device, after which infrared heating is used to activate the enzyme-based reagents for the DNA liberation. The micro-device can be used for STR-typing, including the amelogenin gene (sex typing), although only partial profiles were obtained (21 of 26 alleles expected) [181].

**Figure 6:**
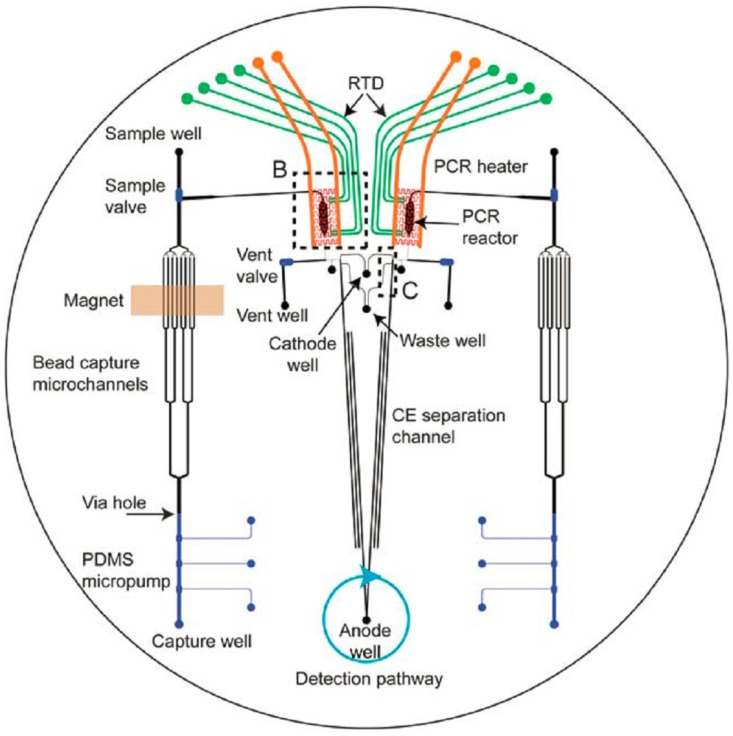
Design of the DNA analysis chip of Liu et al. with sections for DNA purification, PCR and CE separation (reproduced from [179] with permission of The Royal Society of Chemistry).

Most micro-devices are designed for only one of the consecutive steps for forensic DNA analysis. In the last couple of years, several research groups combined two or three of the steps into one chip. Some of the chips do not contain a module for lysis, although off-chip lysis requires an extra transfer step, which increases the risk of contamination. Besides that, often only a fraction of the whole sample is loaded on the chip, and therefore, the PCR might not be successful, as indicated by Lounsbury et al. [181]. Liu et al. have developed a device that combines the steps of PCR amplification and CE [185]. A few years later, they also combined this with purification of the sample and post-PCR cleanup [148]. A nine-plex STR profile from 2.5 ng input standard DNA in about 3 h was acquired with this chip, which is depicted in Figure 6. The step of cell lysis was carried out off-chip [179].

Xu et al. have developed a PCR chip for a forensic test. With the disposable device, short and long DNA fragments could be amplified, as well as STRs. Norland Optical Adhesive 81 (NOA 81) was used as the chip material, which does not absorb DNA polymerase, and therefore, coating (with, e.g., bovine serum albumin) is not required. However, the STR analysis was carried out off-chip with a conventional CE-system [186].

In 2015, Romsos et al. discussed several rapid PCR methods for STR analysis in their review. The PCR protocol has been speeded up by using faster polymerases and by performing direct PCR (i.e., skipping the extraction step). With these adjustments, the cycling time can be brought back to about 45 min (in comparison with 2–3 h for conventional protocols). By miniaturization, the PCR volume is reduced, and the total cycling time can be further brought back. However, most of the devices are specifically designed for (fast) PCR, but do not integrate other steps in the process of DNA analysis [187]. Only a few micro-devices combine the steps from cell lysis up to detection and analysis of the sample, such as the chip of Chen et al. [149]. Lounsbury et al. reported a chip where (part) of a swab can be introduced as input [181]. Integration of all of the consecutive steps in the process of forensic DNA analysis, as described in Figure 1, is still an enormous challenge. However, since for forensic investigations, analysis time and contamination are important issues, the final goal will be to integrate all of these steps in one (micro-)device, which can be used directly at the crime scene.

### 8.2. Commercial Chip-Based Systems

A few companies have developed chip-based systems for forensic DNA analysis, although these commercial systems are still expensive [188].

ParaDNA from LGC Forensics is a system, based on HyBeacons${}^{®}$ technology, that can analyze a crime scene sample within 75 min. The beacons are made of short DNA sequences with one or more fluorescent dyes, which will emit when the complementary DNA is attached to the beacon. ParaDNA screens two STRs and the sex marker amelogenin, and four tests can be run simultaneously. The sample collector takes a small portion of the crime scene sample [189]. Of the 381 DNA samples tested by Ball et al. only 0.15% were discordant with the AmpFISTR^®^ SGM Plus^®^ profile. Dropout by the ParaDNA Intelligence Test was seen for samples with a heterozygote imbalance or due to high stutter [190]. Blackman et al. showed that more then 65% of the samples gave full ParaDNA^®^ profiles (12 alleles) at 4 ng or more DNA (which corresponds to 1 ng per well). For more then 80% of the samples, a “usable” (seven or more alleles detected) profile could be generated with 250 pg of DNA [191].

The RapidHIT™, a machine of 81.5 kg, can create a full STR profile, based on PCR and CE, within 90 min from buccal swabs and other human samples. This system, from IntegenX^®^, contains a cartridge in which five samples (present on a swab) can be run at the same time. A second cartridge contains a positive and a negative control, as well as an allelic ladder [54,192]. Hennessy et al. studied the sensitivity, accuracy and genotype concordance of the RapidHIT system. Upon the addition of DNA to the vials, full profiles were obtained till 500 pg and down to 25 pg 65% of the alleles were detected. With DNA added to swabs, a sensitivity of 100 ng was obtained and 52% of the alleles were detected with an amount of 5 ng added to the swab. All standards tested were in 100% concordance with the certified reference profiles, and for 150 buccal swab samples, a 100% genotype concordance was obtained. The 13 Combined DNA Index System (CODIS) core loci were present on 94.7% of the samples [193]. In the study of Jovanovich et al., a sensitivity level of 176 ng saliva DNA was obtained (concordant full profiles), and still, 57% of the alleles could be detected with 16 ng of DNA on a swab. For the positive controls (37 samples), a 100% success rate was obtained. Two-hundred nineteen of the 250 buccal samples were fully in concordance with the PowerPlex 16 profile on the first pass [54]. At first, the RapidHit was designed for reference samples, such as buccal swabs. Nowadays, a wider variety of samples, such as bloodstains and saliva samples, can be used as input [194]. Verheij et al. showed that DNA profiling is possible when buccal swabs are used, and with the updated system, also saliva, semen, skin and hair samples can be utilized. However, for traces other than buccal swabs, profiling success rates are variable, and with lower input samples, profiling artifacts were present. In their study, blood presumably contained inhibitors, since no profile could be obtained [195].

NetBio has developed a similar system, which uses a BioChipSet™ cassette, to perform STR analysis of 15 loci within 84 min. Five buccal swabs can be analyzed at the same time, and the DNA is purified by guanidinium-based lysis and silica binding. The PCR reaction mix is lyophilized, and the profile is generated by electrophoresis. Of the 100 buccal samples tested, 85 generated a full CODIS profile. Five samples resulted in a partial profile, and from ten samples, no profile could be generated. Allelic concordance was greater than 99.95% (including a spike that was designated as an allele). The failures are caused by blocked channels, resulting in no amplification or electrophoresis of the sample [53].

Furthermore, NEC developed a “portable” DNA analyzer, which weighs 32 kg, and the company claims that it can perform full analysis (nine loci) in only 25 min [196]. However, the entire process, from input to output, takes up to 50 min, with 5 min for DNA extraction, 40 min for PCR amplification and 5 min for CE. Blood samples or buccal swabs can be used as input [197].

Although the chips developed at the research level integrate several steps in the process of DNA analysis in a truly microfluidic manner in a single device, the same level of integration is usually not reached for other components in the system. For example, off-chip electronic circuitry for temperature control or a microscope with a laser setup for detection are required. Therefore, these microfluidic devices cannot yet be operated outside a laboratory environment. In contrast, the commercial systems can be brought to the crime scene, although they are not really portable, considering their weight. Most importantly, however, forensic investigators have limited time on a crime scene and do not always have the opportunity to carry out an analysis that takes up to 75–90 min.

For most commercial systems, the first validation studies are reported in terms of sensitivity, accuracy and genotype concordance and, sometimes, also for robustness (e.g., the use of degraded samples or the presence of inhibitors). In contrast, in the case of research chips, little to nothing is published about the performances in terms of allelic drop-in or drop-out, stutter, background signal or preferential amplification in association with small volumes (and thereby, low quantities of input DNA).

## 9. Outlook

In the last decade, significant progress has been made in the analysis of biological fluids in micro-devices. Single task chips are developed for the purification of genetic material, PCR amplification and STR analysis. Most of the devices are made for only one of the steps of the process of DNA analysis, and only a few have combined several steps in one chip. Besides that, devices that are designed for several steps are often not tested with real forensic samples.

The majority of the microfluidic devices uses a biological sample in a solution or the lysate/pure DNA as input material. The commercially available machines require swabs as the input; nevertheless, integration of trace sampling (e.g., by swabs) on-chip remains difficult and is therefore not yet realized at the research level, thereby becoming one of the technological challenges of DNA analysis on-chip. More research is needed concerning swabbing and sampling techniques that are designed and optimized to be interfaced with chip-based DNA analysis. Various conventional lysis methods are translated to on-chip applications. To make this technique more suitable for forensic samples, single cell lysis might be the way to go. The sample work-up step on-chip can be performed by (μ)SPE or magnetic beads. Crime scene samples often contain low amounts of DNA, and therefore, it is a challenge to obtain as much DNA as possible after the sample work-up upon which amplification can be carried out. To speed up the analysis and improve the limit of detection, amplification can be performed in water-in-oil droplets in microchannels; each droplet functions as an independent reactor with a volume of pico- to nano-liters. To further reduce the analysis time, isothermal amplification is worth investigating. Therewith, instead of cooling and heating rates, as in conventional PCR, the enzyme reaction rate becomes the limiting factor. The (STR) analysis of the DNA fragments is usually performed by a combination of CE separation and fluorescence detection, which is, however, not always performed on-chip. A fluorescent dye that binds to dsDNA is an inexpensive and simple method for the detection step. In case specificity is required, more costly and complex primers or probes can be used. Secure storage of the sample, to minimize the chance of contamination and to ensure the chain of custody, is not yet realized with conventional techniques, but is a big opportunity for on-chip DNA analysis.

By the use of micro-devices, forensic DNA analysis (e.g., STR profiling) can become much faster and performed at the crime scene. However, at the moment, chips cannot be used directly at a crime scene. The majority of the devices still relies on external (non-portable) equipment. The input of most chips is lysate or already purified genomic material. Some commercial systems use buccal swabs as the input, but are not yet validated for evidence samples from a crime scene (“dirty” samples). Impressive on-chip developments have taken place; nevertheless, there is still no portable device containing all of the required steps available for fast analysis (within about 30 min for a full STR profile) of real “dirty” samples directly at the crime scene. It would also be ideal to have a multi-compartment chip for additional laboratory analyses and the storage of the sample (extract).

The most progress, considering STR profiling, has been made by the discussed commercial systems. Although the developed apparatuses are relatively heavy and therefore not applicable for portable use at the crime scene, they could provide valuable information upon use at a police station. Obtaining a full profile within even less time than possible at the moment is still a great desire of forensic scientists, but speed is not the only challenge within DNA analysis. There is also a desire to obtain a profile from trace samples, which contain a low amount of DNA (less than 100 pg), or degraded samples. Additional challenges with trace DNA are mixed DNA profiles and contamination [50]. These issues have not yet been incorporated within forensic DNA analysis on-chip.

The latest developments concerning DNA analysis on-chip focus on STR profiling. These trends might also contribute to developments within other biological trace research, such as Y-chromosomal profiling, mt-chromosomal profiling, RNA cell typing and phenotype profiling. One of the challenges of chip-technology is to develop platforms that enable other DNA or RNA analysis techniques.

Before micro-devices can truly be applied at a crime scene, some jurisdictive hurdles need to be overcome, as well. Amongst other aspects, the following issues need to be considered: the regulations concerning a forensic expert for the interpretation of DNA profiles and the (Dutch) law states that forensic DNA investigations can only be conducted by an accredited laboratory, the conditions for the uptake of a profile in the DNA databank, the right of contra investigation and the retention period of DNA (extracts). It is unclear at this point whether such regulations will hamper or stimulate the introduction of lab-on-a-chip devices in forensics or whether new regulations need to be established, in order to fully exploit the possibilities offered by micro-devices.

## Figures and Tables

**Table 1:** Overview of various PCR chips. CE, capillary electrophoresis.

Type	Material	Cycles *	Detection	Year and Ref.
Well-based	SU-8	Melting curve experiment 35 (199 bp, 90 min)	SYBR Green (melting curve) Electropherogram (off-chip)	(2004) [56]
	PDMS/Glass (droplet array)	40 (several amplicons, 65 min)	EvaGreen (real-time + melting curve)	(2009) [61]
	Silicon (droplet array)	45 (18–25 bp, 65 min + 66 min) micro RNA, RT-PCR	TaqMan probes (real-time)	(2011) [62]
	Polycarbonate	32 (243 and 96 bp, 45 min)	CE (on-chip)	(2016) [63]
Fixed-loop	Glass	20 (176 bp, 1.5–19 min)	Gel + EtBr (off-chip)	(1998) [64]
	Glass	20, 25, 30, 35 and 40 (230 bp, 17 min for 40 cycles)	SYBR Green (off-chip)	(2003) [65]
	PMMA	20 (990 bp, 57 min)	Gel + EtBr (off-chip)	(2009) [66]
	Pyralux	30 (90 bp, 5 min)	Gel + EtBr (off-chip)	(2014) [67]
	FEPtubing	40 (Plasmid clones and *Escherichia coli* 40 min)	TaqMan probes	(2014) [68]
Closed-loop	Ceramic	40 (209 bp, 27–70 min)	Electronic	(2003) [69]
	Teflon	35 (305 and 700 bp, 73 min)	Gel + EtBr (off-chip)	(2004) [70]
Oscillatory	Silicon/Pyrex	20–30 (only theoretical model)	Gel + EtBr (off-chip)	(2003) [71]
	Silicon	35 (Human papillomavirus, 15 min)	Gel + EtBr (off-chip)	(2005) [72]
	PDMS	12(−20) (Plasmid DNA, 3–4 min)	SYBR Green	(2007) [60]
	PDMS/Glass	30 (Hepatitis B virus, 23 min)	TaqMan probe	(2014) [73]

* Cycle time including initial denaturation and final extension, if used. If no amplification time were given, the total time of the PCR protocol was taken.

**Table 2:** Overview of various droplet PCR chips.

Type	Material	Cycles *	Droplet Size	Detection	Year and Ref.
T-junction	Silicon/Pyrex	40 (unknown amplicon, 108 min)	8–15 pL	FAM	(2007) [85]
	Polycarbonate	32 (60 bp, 14–19 min)	100–155 m	Fluorescence	(2007) [86]
	SU-8/PMMA	34 (85 bp, 17 min)	131 pL	Gel + SYBR Green (off-chip)	(2009) [87]
Flow focus	PDMS/Glass	34 (245 bp, 35 min)	65 pL	FAM and Alexa Fluor 594	(2008) [88]
	Glass	25 (101 bp, 46 min)	3 nL (agarose)	SYBR Green (off-chip)	(2010) [89]
	PDMS	40–45 (150–300 bp, 30–90 min)	50 pL	FAM	(2011) [90]
	Glass	25 (several amplicons, 46 min)	pL (agarose)	SYBR Green (off-chip)	(2012) [91]
	PDMS/Glass	32 (STR, 152 min)	nL (agarose)	CE (off-chip)	(2014) [92]

* Cycle time including initial denaturation and final extension, if used. If no amplification time were given, the total time of the PCR protocol was taken.

**Table 3:** Overview of various isothermal amplification methods. LAMP, loop-mediated isothermal amplification; MDA, multiple displacement amplification; HDA, helicase-dependent isothermal DNA amplification; RCA, rolling circle amplification; SDA, strand displacement amplification.

Method	Polymerase	Temperature	Primers	Speed/Yield	Remarks
LAMP [106,107]	Bst	60–65 ${}^{\circ }$C	2 or 3 sets	10–20 g in 30–60 min 10${}^{9}$ copies < 1 h 3-fold every half cycle	Highly specific Detection by turbidity Complex primer design
MDA [108,109]	φ29	30 ${}^{\circ }$C	Random hexamers	Exponential amplification	High processivity Direct amplification of lysate (no purification)
HDA [110,111,112]	Helicase	37 ${}^{\circ }$C (mesophilic) 60–65 ${}^{\circ }$C (thermophilic)	1 set	10 ng from 10${}^{3}$ copies Exponential amplification	UvrD helicase has limited speed and processivity Helimerase is more efficient
RCA[103,113,114]	φ29 Klenow	37 ${}^{\circ }$C	1 or 2 primers	53 nucleotides/s 70 kbp in 20 min	Circular template needed Can be used with padlock probes
SDA [115]	exo${}^{-}$ Klenow	37 ${}^{\circ }$C	1 set	Exponential amplification 10${}^{10}$-fold 10${}^{6}$-fold after 5 h	Inefficient at long amplicons Denaturation needed Complex primer design

**Table 4:** Overview of various isothermal amplification chips. RPA, recombinase polymerase amplification.

Method	Material	Amplicon	Volume	Detection	Year and Ref.
LAMP	Silicon	Virulence genes (various, 20 min)	50 μL (10 chambers)	Turbidity and SYBR Green	(2011) [116]
	PDMS	*λ*DNA (48,502 bp input, 70 min)	2 μL (for nL droplets)	Calcein	(2012) [117]
	PDMS/Glass	Virulence genes (various, 60 min)	30 nL droplets	EvaGreen	(2013) [118]
	PMMA	Pathogenic bacteria (various, 30 min)	48 μL (1.414 μL/reaction well)	Gel + SYBR Green (off-chip)	(2014) [119]
	PMMA	*Salmonella* (-, 70 min)	25 μL	SYBR Green	(2016) [120]
MDA	PDMS	*E. coli* (whole genome, 10–16 h)	60 nL	SYBR Green (off-chip)	(2007) [121]
	PDMS	MCF-7 cells (whole genome, 16 h)	1.4 nL	qPCR (off-chip)	(2015) [122]
HDA	Polymeric	BRCA1 gene (113 and 157 bp, 15 min)	35 μL	Gel	(2015) [123]
RCA	Glass	16 S rDNA (various, 110 min) off-chip amplification	30 μL	SPR-Biosensor	(2014) [124]
	Glass	OLR1 gene (-, 30 min)	500 nL	EvaGreen	(2015) [125]
RPA	Various	*E. coli* (bla${}_{CTX-M-15}$ gene, 15 min)	270 nL	Cy5 labeled probes	(2015) [126]
	DVD-R discs	Various (-, 2 h)	3 μL (sample volume)	Optical density	(2016) [127]
	PMMA/Glass	2 viruses and 1 bacterium (142, 144 and 181 bp, 48 min)	54 μL	Luminol	(2016) [128]

**Table 5:** Overview of various integrated chips. SPE, solid phase extraction; AD, amperometric detector.

Chip Material	Lysis	Extraction	Cycles *	Detection	Remarks	Year and Ref.
Glass	-	-	PCR 20 (136 bp, 10 min)	CE	280 nL PCR chambers Valve design	(2000) [177]
PDMS/Glass	Thermal 2 min @ 95 ${}^{\circ }$C	-	PCR 30 (273 bp, 27 min)	Gel + EtBr Off-chip detection	EOFpumping	(2005) [11]
PMMA + silicon + PDMS/Glass	Chemical Guanidine lysis buffer	SPE Porous silicon	PCR 35 (293 bp, 50 min)	SYBR Green and Gel	Region for cell separation Blood sample	(2007) [178]
Polycarbonate	Chemical Lysis buffer	SPE Silica membrane	(RT-)PCR 25–35 (Bacterial/viral, 28–130 min)	Up-converting phosphor reporter particles Antibody-antigen	Paraffin valves Dried reagents in the chip	(2010) [149]
Borofloat glass	-	SPE Silica beads	PCR 28 (STR, 140 min)	-	STR fragments Off-chip detection	(2010) [47]
Borofloat glass	-	SPE Silica beads	PCR 32 (STR, 60 min)	-	STR fragments Off-chip detection	(2011) [48]
Glass + PDMS	-	Beads Magnetic	PCR 32 (STR, 40 min)	CE Biotin label	STR fragments Resistance temperature detector	(2011) [179]
Glass + PDMS	Electrochemical 0–10 V DC	-	PCR 25 (100–595 bp, -)	CE-AD and Gel + EtBr	Amperometric detection Combination of modules	(2012) [180]
PMMA + PDMS	DNA liberation Enzymatic	-	PCR 32 (STR, 26 min)	-	STR fragments IR heating	(2013) [181]
PDMS/Glass	-	Membrane Aluminum oxide	PCR 60 (Bacterial, 101 min)	FAM probes	2-step PCR	(2013) [182]
Cyclic olefin polymer	-	-	PCR 27 (STR, 45 min)	Electrophoretic PowerPlex	STR fragments For reference samples	(2014) [150]

* Cycle time including initial denaturation and final extension, if used. If no amplification time were given, the total time of the PCR protocol was taken.
